# Anxiety relief in the post-pandemic era: a randomized trial on the integration of digital technology into dance art healing

**DOI:** 10.3389/fpsyg.2025.1545461

**Published:** 2025-05-22

**Authors:** Suhui Qiu, Chao Ruan, Ying Wang

**Affiliations:** Art and Design Institute, Zhejiang Sci-Tech University, Hangzhou, China

**Keywords:** post-pandemic era, anxiety, dance, art healing, digital technology, somatosensory interaction

## Abstract

**Trial design:**

This randomized controlled trial examined the effectiveness of digital technology-integrated dance therapy in alleviating anxiety symptoms associated with post-pandemic isolation.

**Methods:**

Participants, including both professional and amateur dancers, were randomly assigned to either a traditional dance therapy group or a digital dance therapy group utilizing smart fitness mirrors. Anxiety levels were assessed using the Positive and Negative Affect Schedule (PANAS) before and after the intervention and a semi-structured interview was conducted. The trial spanned 3 months, with participants engaging in structured dance therapy sessions twice a week.

**Results:**

The findings revealed that digital dance therapy led to a significant improvement in overall anxiety reduction, as indicated by enhanced PANAS scores post-intervention. Participants in the digital dance group exhibited a notable increase in positive emotions, whereas reductions in negative emotions were less pronounced and, in some cases, even showed a slight increase. Approximately half of the participants experienced a significant decrease in anxiety symptoms, with the digital intervention demonstrating greater effectiveness compared to traditional dance therapy. Additionally, qualitative feedback indicated widespread acceptance of digital dance tools, with participants recognizing their potential in alleviating social and body image anxiety.

**Conclusion:**

While digital dance therapy shows promise in enhancing positive emotions and reducing anxiety, its effectiveness in addressing negative emotions remains inconclusive. The study highlights the need for extended intervention periods, larger sample sizes, and further refinement of digital tools to optimize therapeutic outcomes. Future research should explore long-term efficacy and improve human-computer interaction in digital dance therapy.

## 1 Introduction

In the post-pandemic era, global society has undergone an unprecedented transformation. The public health crisis triggered by the COVID-19 pandemic has not only reshaped people’s lifestyles and work patterns but has also profoundly affected their psychological well-being. Research has shown that multiple factors—such as social isolation, economic stress, health concerns, and uncertainty about the future—brought about by COVID-19 have led to a complex and serious psychosocial situation (Smith et al., 2020). The World Health Organization has recognized that such self-isolation/social distancing measures may result in individuals becoming more anxious, angry, stressed, agitated, and withdrawn (World Health Organization, 2020). Among these, anxiety is particularly prominent. Anxiety is one of the most prevalent mental health disorders (Bandelow and Michaelis, 2015) and is defined as a persistent feeling of worry, fear, or nervousness (Mental Health, 2020), making it a significant mental health challenge in the post-pandemic era. The pervasive feelings of isolation, helplessness, and disruption to daily life have exacerbated anxiety, posing a serious threat to individuals’ mental health (Wang et al., 2021). The UN E-Government Survey (Vereinte Nationen, 2020) assessed the digital transformation of governments during the pandemic and found that digitalization demonstrated strong adaptability and resilience. Digital tools, such as social media and videoconferencing, enabled people to stay socially connected during quarantine, facilitating social interactions and alleviating psychological stress. The World Health Organization (Ting et al., 2020) has also issued guidance stating that digital healthcare services, such as online consultations and telemedicine, have improved access to healthcare services during the pandemic.

In this context, it is particularly important to explore ways to effectively alleviate anxiety. Art therapy is a healing process when art forms act as a medium in the field of psychotherapy (Huili et al., 2023). Research on art healing in the last decade has shown that art and cultural engagement have a positive impact on people’s health (Howarth, 2018). Abbing et al. (2018) used a Cochrane analysis to explore the effectiveness of art therapy for adult anxiety disorders, but it did not conclude the effectiveness of AT for adult anxiety disorders, and the experimental approach of the study needs to be updated to be consistent with the study of anxiety in people’s lifestyles in the post-pandemic era. Ni and Hu (2012) concluded in her 2012 review that most Chinese researchers are committed to reducing mental pain and alleviating depression and anxiety in post-disaster patients. The study suggests that there is still a lot of room for the development of art therapy in China in the fields of education and healthcare, and the development of information technology will also have an impact on the art therapy model. In light of these developments, the necessity of exploring online art-based therapy became increasingly apparent. Online therapy not only offered a viable alternative for those unable to access in-person sessions but also leveraged digital technology to provide new avenues for psychological intervention.

Digital technology, with its convenience, accessibility, and ability to offer personalized experiences, has become a key tool in the realm of mental health services. It transcends geographical barriers, enabling interventions for mental health conditions such as anxiety. Argo et al. (2014) demonstrated the potential of such technology through their multi-sensory exposure therapy, which combined sound, music, and visual stimuli to create an immersive experience for patients with anxiety (Argo et al., 2014). This digital therapy allowed patients to engage in psychological treatment from the comfort of their homes, alleviating anxiety-driven avoidance behaviors. Thus, online art-based therapies are not merely a temporary solution during the pandemic but represent a promising direction for the future of mental health services. Huili et al. (2023) and others integrated somatosensory interaction technology into picture books in their study in 2023, designed a somatosensory interactive game for a group of children with autism, and verified that somatosensory game therapy and traditional picture book therapy have their own advantages. However, research on somatosensory interactive digital devices for anxiety and other psychological conditions in China remains scarce, with even less focus on integrating such technology into dance therapy. The rapid development of digital technology has also brought more new forms of dance, Kinect’s real-time capture of dancers’ movements, Musk’s robotic coaches, and GPT-4’s personalized plan generation and customization have all made the intervention of digital AI coaches at the level of personal health and life an inevitable trend.

As a form of art therapy, dance art healing not only focuses on the movement and expression of the body but also emphasizes the feelings and connections of the mind. It helps individuals enhance their self-awareness, thereby achieving the goals of reducing stress, relieving anxiety, and promoting mental health (Christensen et al., 2021). Koch et al. (2019) demonstrated in a 2019 meta-analysis that DMT and dance interventions can improve clinical outcomes, cognitive outcomes, and (psycho)motor outcomes. Her study included both group and individual therapy sessions, based on the development of dancers or movement coaches from diverse backgrounds. Karkou et al. (2019) used qualitative meta-analysis and quantitative meta-analysis for people aged 16 to 65 years to put together a systematic review of studies on the effectiveness of DMT for people with depression. A systematic review of the studies was conducted and the results showed that DMT alone can have a positive impact on patients with a primary diagnosis of depression, but with 81% of its participants being female, this study has some gender limitations. Neither of these studies categorized the volunteer dancers by professional status (i.e., professional dancers versus amateur dancers). The present study, however, took into account the professional nature of dance, where varying degrees of dance technique may affect the release of anxiety. As demonstrated by subsequent experiments, professionalism was not an influencing factor (*p* > 0.05).

In the spring of 2020, the outbreak of COVID-19 led to the closure of dance studios and other in-person therapeutic spaces worldwide. As a result, many traditional forms of psychological interventions, such as dance therapy, were forced to pause, and mental health issues— particularly body image anxiety and social anxiety caused by the isolation of the pandemic— surged. Adilogullari (2014) used a quantitative scale to study the effects of a 12-week Latin dance workout on social body image anxiety and concluded that dance was effective in reducing social anxiety, but his study did not mention the factor of body changes. Karkou et al. (2019) summarized that, in all studies, scales measuring depression severity have been found to be sensitive to capturing mood, body image, health anxiety, and many other factors related to the diagnostic criteria for depression. However, Karkou did not suggest strategies to alleviate such anxiety. There is limited research on dance movement therapies for relieving body image anxiety and social anxiety. This study, therefore, explores effective dance movement therapies for relieving body image anxiety and social anxiety, taking into account the legacy of anxiety from the pandemic.

Therefore, this study aims to explore how digital technology, especially somatic interactive smart devices, can alleviate anxiety through dance art healing in the post-pandemic era. Specifically, we will focus on how digital platforms can support the development of remote dance art healing activities and adopt a mixed research approach, i.e., a combination of psychological scales, questionnaires, and semi-structured interviews, to analyze the mechanism by which it enhances participation, therapeutic effects, and expands the scope of benefits. At the same time, we will also explore how digital technology can be combined with the characteristics of dance art healing to design personalized, interactive, and enjoyable intervention programs to better meet the mental health needs of different populations.

The significance of this study lies in the fact that, on the one hand, it enriches the means of intervention in the field of mental health and provides new ideas and methods for alleviating anxiety and other psychological problems. On the other hand, it promotes the application and development of digital art in the field of mental health and demonstrates the great potential of the fusion of science and technology and humanities in promoting human mental health. Through this study, we expect to provide a scientific basis and practical guidance for effectively intervening in anxiety problems in the post-pandemic era and, at the same time, lay the foundation for further exploration and application of digital art in the field of mental health.

## 2 Dance movement therapy

Dance Movement Therapy (DMT) is a therapeutic form that takes place in well-controlled clinical settings for the purposes of therapy and personal growth (Christensen et al., 2021).

The historical development of Dance Movement Therapy (DMT), as a therapy that combines dance with psychotherapy, can be divided into three professionally focused phases: emotional body/movement, body/mind, and social/relationships (Miller, 2016). The birth of DMT is closely linked to the development of modern dance, and many of the pioneers of modern dance made important contributions to the early development of DMT. Marian Chace, a key figure in the establishment and development of DMT—recognized as one of the founders of modern dance therapy—was invited to join the staff of St. Elizabeth’s Hospital in Washington, DC in 1942. Her work laid the foundational practice for integrating dance and the therapeutic process, greatly influencing the field of DMT (Bunney, 2013). Another important early dance therapy pioneer, Mary Whitehouse, emphasized the role of dance in personal growth and self-expression, and, along with Chase, contributed to the early development of DMT (Bunney, 2013).

Rudolf Laban began to link the body to the mind, emphasizing the interplay between physical movement and mental processes. His system of Movement Analysis (Laban Movement Analysis) provided a scientific tool for observation and analysis, making dance therapy more systematic and specialized (Karkou et al., 2019). In 1916, Carl Gustav Jung documented the idea of dance as a psychotherapeutic treatment (Chodorow, 2013). Jung’s analytical psychology emphasized the importance of the unconscious and symbols in psychotherapy, and his theories provided deep theoretical underpinnings for DMT, helping visitors explore the unconscious within. Wilhelm Reich’s theory of bioenergetics emphasizes the interaction between the body and the psyche, and he believes that physical tension is closely related to psychological conflict. His theory provides DMT with a theoretical basis for exploring psychological issues on a physical level (Feldman, 2015).

The use of DMT as an intervention to enhance emotional regulation and control has attracted increasing attention in applied research in psychology and therapy. DMT can help individuals improve body image, reduce stress, and enhance emotional, cognitive, social, and physical integration (Berrol, 1990). This aligns with efforts to address body image anxiety and social anxiety triggered by the isolation during the COVID-19 pandemic (Zhang et al., 2020; Swami et al., 2021). Röhricht and Priebe (2006) applied dance therapy to the treatment of patients with schizophrenia and achieved significant results, providing important evidence for the use of DMT in the treatment of mental illness. Judith Kupfer applied dance therapy to the treatment of post-traumatic stress disorder and developed a corresponding treatment model. This not only expanded the application of DMT but also demonstrated its effectiveness in treating trauma. Mary Ann Cleveland, on the other hand, made an important contribution to the development of dance therapy for children and adolescents by using it to help them deal with emotional problems and build relationships. This shows that DMT can be applied in a variety of settings such as mental health, medical, educational and community (Koch et al., 2014).

Dance has primarily been viewed as a form of recreation or entertainment, with little role in healing. However, there have been significant developments in the health applications of dance over the past 40 years, and growing evidence and recognition of its therapeutic benefits of dance in disease prevention and rehabilitation (Kshtriya et al., 2015). Recent studies have highlighted the therapeutic potential of dance, particularly in the context of Parkinson’s disease (PD). Morris and Slade investigated the feasibility and impact of online dance therapy on consumer engagement among individuals with PD (Morris et al., 2021). Their research demonstrated that engaging in online dance sessions not only enhanced participant engagement but also contributed positively to their emotional well-being. Similarly, Bek et al. (2025) explored the challenges and opportunities associated with digital dance programs for PD patients. Their findings underscore the efficacy of dance as a multimodal intervention that integrates mental, musculoskeletal, and neurological processes. This integration can lead to improvements in various domains, including procedural learning, attention, memory, coordination, rhythm, balance, and gait. Notably, dance therapy has shown promise in addressing cognitive, emotional, and neurological disorders prevalent among PD patients. These studies collectively reinforce the growing recognition of dance as a valuable therapeutic tool in disease prevention and rehabilitation, particularly for individuals with neurological impairments such as Parkinson’s disease.

Kshtriya’s extremely team conducted a comprehensive literature search for different dance interventions using six different databases, primarily using literature analysis and review, to critically review the existing literature on the neurological effects of dance interventions (Kshtriya et al., 2015). While the study provides a rich overview and theoretical analysis, further research is needed regarding the direct effects of dance on neurobiological factors.

A systematic review conducted by Koch et al. (2019) evaluated the effects of dance and dance movement therapy (DMT) on health-related psychological outcomes. The review identified 41 studies (dance interventions: *N* = 20; DMT: *N* = 21) published between January 2012 and March 2018, encompassing a total of 2,374 participants. The authors noted that the difference in population made comparisons difficult because most dance intervention studies came from preventive contexts, and most DMT studies came from institutional healthcare contexts with more severely impaired clinical patients. This study suggested that DMT and dance interventions impacted different domains: DMT decreased depression and anxiety and increased quality of life, interpersonal and cognitive skills, whereas dance interventions increased (psycho-)motor skills.

A meta-analysis evaluated the effectiveness of dance movement therapy (DMT) and dance interventions for psychological health outcomes. Results suggest that DMT decreases depression and anxiety and increases quality of life and interpersonal and cognitive skills, whereas dance interventions increase (psycho-)motor skills. Most dance intervention studies came from preventive contexts, and most DMT studies came from institutional healthcare contexts with more severely impaired clinical patients, where there were smaller effects (Cox and Youmans-Jones, 2023). This distinction highlights the need for tailored therapeutic approaches, especially as we explore how digital tools can enhance both preventive and clinical applications of art-based therapies.

The authors of this article also believe that dance can help individuals recover from clinical levels of dysfunction. However, different clinical populations would likely benefit from different dance practices. Thus, using hobby dances (e.g., Argentine tango, ballet, ballroom, hip hop, etc.) as a therapy for clinical conditions (cancer, trauma, Parkinson, dementia, ADHD, etc.) still requires substantial empirical support from research in healthy populations to formulate clear hypotheses about why the choice of one specific dance style (and not another) might be beneficial for a specific health problem. Such targeted assessments are currently not common practice, and researchers often treat “dance” as a single, unified entity, which it is not. This heterogeneity makes the results appear inconclusive and piecemeal, deterring policymakers from making targeted investments into the health effects of dance practice as a recreational activity with important psychophysiological and health benefits.

## 3 Problems of anxiety caused by COVID-19

A study conducted a large-scale survey of psychological distress in the Chinese general population during the COVID-19 period. The results demonstrated the incidence and severity of psychological distress brought about by COVID-19. Society is currently burdened with a high level of mental health issues, which is specifically characterized by body image anxiety and social fear. These findings provides a concrete basis for the development and implementation of relevant mental health intervention policies to address this challenge efficiently and effectively (Qiu et al., 2020).

### 3.1 Body anxiety

The stress and anxiety caused by the coronavirus (COVID-19) pandemic pose a serious threat to the mental health of the global population, and, by extension, to body image outcomes. Some pre-pandemic research—mostly involving samples of undergraduate women—has shown that perceived stress (i.e., a person’s appraisal of stress caused by environmental conditions) and stressful life events were associated with greater body dissatisfaction [e.g., (Haddad et al., 2019; Johnson and Wardle, 2005; Murray et al., 2011)]. Stratified regression results indicated that COVID-19-related stress and anxiety are associated with more negative body image, over-and-above-perceived stress, stressful life events, and trait anxiety (Swami et al., 2021). It is possible that COVID19-related stress and anxiety diminish coping resources to manage threats to body image, increase exposure to thin/athletic ideals via media messaging (e.g., given increased screen time under lockdown; see Pietrobelli et al., 2020), and heighten concerns about weight and/or shape changes that occur during conditions of lockdown (e.g., because of decreased physical activity) (Cooper et al., 2022; Rodgers et al., 2020). COVID-19-related stress may also be associated with a greater frequency of negative body ruminations that lead to a preoccupation with body shape and/or weight, as well as a desire to reassert a degree of control through bodywork (Ruggiero et al., 2008). Women have adopted unhealthy weight control behaviors to manage their weight, thereby moderating the body image anxiety associated with the COVID-19 pandemic; whereas men seek to reaffirm a sense of control and increase their masculine capital through a desire for stronger muscles and contemplation of perceived body image (Swami et al., 2021). Efforts to address negative body image under lockdown conditions will require new palliative interventions (e.g., telemedicine, and guided self-help interventions) (Cooper et al., 2022).

### 3.2 Social anxiety disorder

The confinement situation during the COVID-19 pandemic is not really an “enclosed space” but more related to the reduced daily activities. The majority of people were ordered to stay at home for long periods and work remotely. This resulted in reduced physical activity, which negatively impacts mental health in the community, as physical activity directly reduces general negative emotions (Zhang et al., 2020). This isolation led to increased stress, which caused anxiety that may have developed into coronophobia, related to the avoidance of public places and events—social anxiety disorder (SAD) (Amin, 2020; Lee et al., 2020).

Among anxiety disorders, social anxiety disorder, characterized by a severe fear of social interactions and attention, which the core experience being an extreme dread of judgment and negative evaluation by others, has been exacerbated by the pandemic’s constraints. It is often accompanied by a heightened awareness of perceived flaws or failings (American Psychiatric Association, 2013). Individuals with higher social phobia tend to pay more attention to themselves than to others in social situations, this self-focus exacerbates anxiety (Clark and Wells, 1995).

Some studies have shown that SAD has been exacerbated by the pandemic’s constraints (Xia, 2024). COVID-19-induced online environments can amplify other-focused perspectives. Since people are often required to pay closer attention to others’ thoughts and feelings without direct physical interactions, many people might have become more concerned about others’ potentially negative responses because of the avoidance of social situations (Tei and Wu, 2021). People are concerned about their value in the eyes of others, as well as the misery they may cause others due to quarantine or societal rejection (i.e., fear of infecting or troubling others, in addition to dread of being infected). Furthermore, many people are still concerned that their COVID-19 status will be leaked to others. Meanwhile, according to the Compensatory Internet Use Theory of Internet Use Disorder (IUD) (Dieris-Hirche et al., 2023), Internet use serves as a means to alleviate negative emotions. However, excessive Internet users may also be more susceptible to the rapid dissemination of misinformation and unfounded fears through online media.

People expect art healing to provide emotional relief, self-expression, and therapeutic benefits, especially during times of stress or crisis. Engaging with art—whether through creating or observing—offers individuals a means of processing complex emotions, reducing anxiety, and fostering a sense of well-being (Daniel, 2021). During times of collective trauma, such as COVID-19, people have shown a high level of motivation to heal or alleviate anxious psychological issues through this art-based healing, through online dance (Rugh et al., 2024).

## 4 Healing mechanisms based on body perception in digital art

### 4.1 Digital technology in art healing

Body-awareness-enhancing and body-centered therapies are gaining popularity. These methods include yoga, tai chi, mindfulness therapy, meditation, body-oriented psychotherapy, etc. Mehling argued that “body awareness is a complex, multidimensional concept that requires a more nuanced conceptualization” (Mehling et al., 2009).

With the rise of digitalization, there is more room for art healing. These technologies include, but are not limited to, the following currently popular forms: apps, various types of image creation and film editing software, animation, gaming, virtual reality (VR), and participatory environments, tablet technology, light painting, artificial intelligence, digital storytelling, and other technological media. There are two main applications of digital technology in somatic art healing: art therapy using somatic tools and art creation using digital media during therapy (Zubala et al., 2021).

The use of online somatic tools for art therapy is particularly well-suited to teletherapy, a field of cognitive-behavioral therapy conducted online that allows human cognitive behaviors as well as bodily senses to be embodied in symbolic, metaphorical, and projective artistic methods through any medium. For example, Tsinghua University scholars have created a brain-machine-painted dream based on real-time EEG analysis and algorithmic design, which visualizes and analyzes brainwave signals for different needs. It can be used for health management, sleep monitoring, and healing services for perceptually impaired groups.

The use of digital technology in art therapy is not limited to online communication tools but extends to the application of digital media for the purpose of art-making. A challenge identified in the early stages of discussions on the use of technology in art therapy was the need for increased collaboration between art therapists, designers and developers in order to devise technological solutions suitable for art therapy practice (Gussak and James, 1999). Limited attempts to develop art therapy-specific electronic devices to date have lacked in-depth input from art therapists at the technical stage, and, consequently, there has been inappropriate integration of the established processes of art therapy with technology (Mihailidis et al., 2010; Mattson, 2015). In effect, art therapists who incorporate digital arts media into their practice elect to use painting apps that are not necessarily suitable for art therapy practice. Therefore, how digital technology can be integrated into art healing in teletherapy has yet to be further explored.

### 4.2 Main tools and platforms

Technology and science have long been devoted to exploring their ability to visualize the complexities of human experience, physical properties, and human communication, and dance has maintained an important relationship in its participation in these developments. Increasingly, dance works transform information about dance movement, form, and spatial location into digital signals that are stored, processed, and presented through computer technology. This digitization process can be realized with the help of a variety of technological tools, including motion capture technology, virtual reality, augmented reality, artificial intelligence, etc. (Whatley and Varney, 2009).

There are significant differences between dance under digital technology and real dance in terms of presentation, interactivity, and dissemination (Anker, 2020). Digital technology preserves dance as a digital file, which can be played repeatedly and is easy to modify. In terms of dissemination, digital dance is mostly disseminated in virtual spaces such as online platforms, and has a wider range of dissemination compared to traditional dance forms like live performances and recordings. Through specific devices, digital dance enhances interactivity. From the perspective of perceptual visualism proposed by Don Ihde, digital technology provides the dancer with an extension of the body that is not limited by space and time, allowing the body to remain “present” in an “absent” way (Strutt and Cisneros, 2021; Song and Dong, 2021).

In summary, we have divided dance healing with digital technologies that exist today into two categories: dance healing using somatic digital tools and dance creation using digital media during therapy.

**Figure 1:**
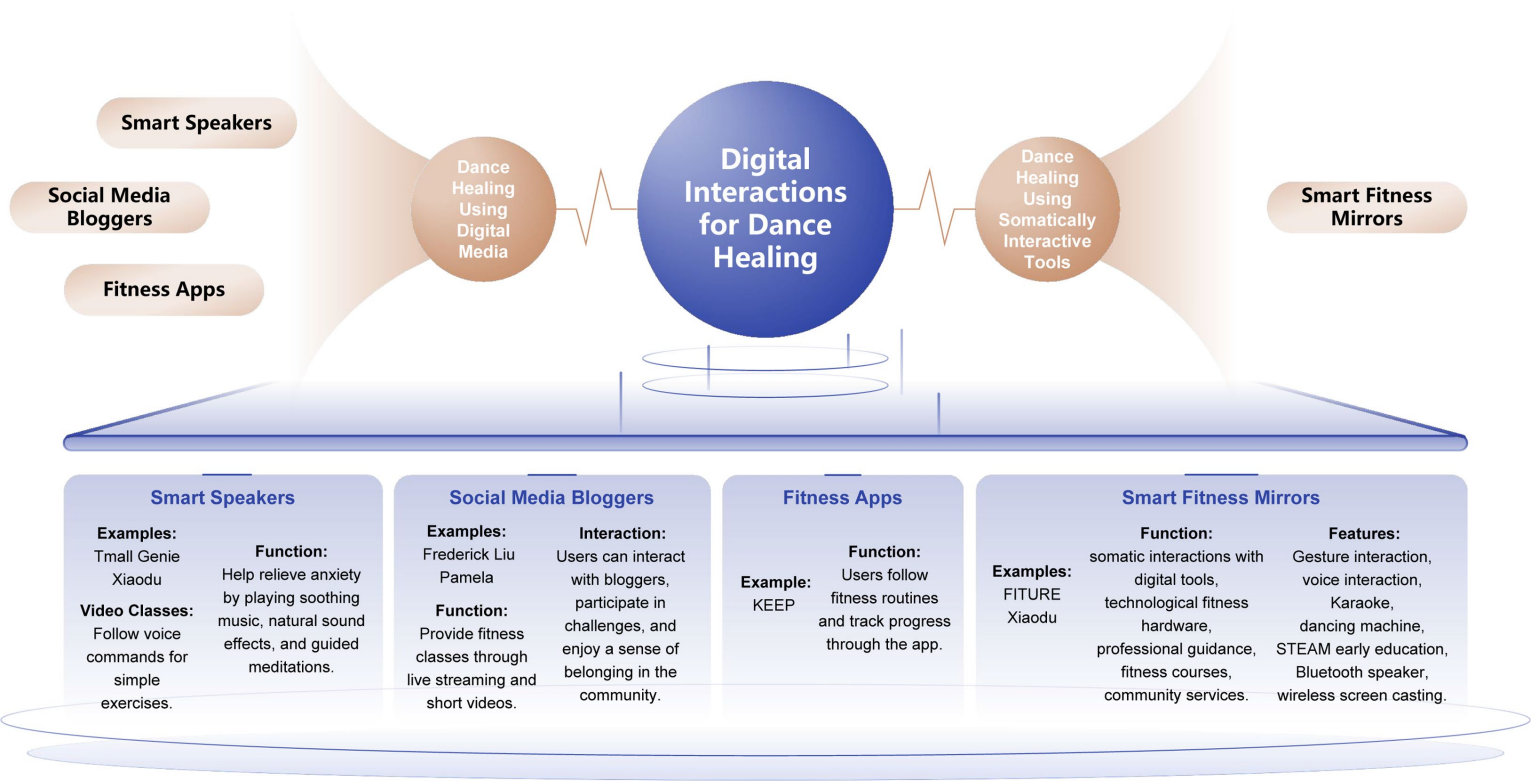
Digital tools to assist in healing realization.

During the quarantine phase of the pandemic, digital technologies were utilized for self-management as well as physical healing to stay physically active at home. Key examples of the use of digital interactions during the pandemic included smart speakers (e.g., Tmall Genie, Xiaodu), tracking of physical activity content on media platforms such as Bilibili (following netizens such as Frederick Liu and Pamela) (Sun et al., 2020), use of fitness apps (e.g., KEEP), and use of smart fitness mirrors (e.g., FITURE, Xiaodu, etc.) (Yu et al., 2022; Zhong et al., 2020). The first three are examples of dance healing using digital media, while the latter involves dance healing using somatic interaction digital tools (see Figure 1 for details). Somatosensory interaction with the help of digital technologies such as motion capture, emotion detection, and virtual reality allows for the digitization of the body’s movements and perceptions. By amplifying subtle changes in the body, somatosensory technology enables participants to see their real-time status and enter a positive feedback loop (Chang et al., 2023).

In somatosensory interactive art healing installations, such as smart fitness mirrors, the designer employs the theories of psychological Gestalt in Merleau-Ponty’s *Phenomenology of the Body*. For example, the influence of the visual senses on the body is utilized to provide the psychological catharsis necessary for regulation of anxiety in young people.

Table 1 aims to explore how emotional problems caused by the COVID-19 pandemic can be alleviated through digital somatosensory interactive products. Based on an analysis of the causes of anxiety in young people, combined with psychotherapeutic theories, the table proposes specific design requirements and strategies aimed at optimizing the use of digital somatosensory devices at home. In particular, by discussing smart devices suitable for home use, Table 1 emphasizes the role of these products in promoting physical activity and social interaction, helping users to reduce the anxiety and stress generated during the quarantine period of the pandemic.

**Table 1:** Theoretical foundations and strategies for mitigating emotional problems with digital somatic interaction products.

Construct reasons	Theoretical basis	Construct elements	Appeal or strategy
Lack of physical activity in the home due to isolation from the pandemic may cause body image problems.	The relationship between digital somatic interaction products and the likelihood of people participating in home-based exercise.	Digital somatosensory interactive devices suitable for home use should have intelligent feedback features.	Clarify the specific smart digital somatosensory interactive products to be used and specify the time of use.
Isolation from the pandemic has reduced the need for socialization, leading to social anxiety and emotional distress in some populations.	Dance healing, as well as a comfortable environment, are important factors in alleviating social fears and addressing emotional stability.	The home environment should provide space and equipment suitable for dance practice and take into account the individual needs of the user.	Groups or professional dancers affected by the pandemic quarantine, with dance hobbies, experience, or professional backgrounds, were selected to use the products at home and to track the process and results of their use.

## 5 Method

### 5.1 Participants and procedures

This study was carried out with a randomized controlled experimental design. A total of 200 participants were recruited for this study using convenience sampling, a type of non-probability sampling, through open recruitment via social media and existing contacts. Following the Self-Assessment Scale for Anxiety (SAS) psychological assessment, 152 subjects were excluded, and 48 participants were ultimately selected for the study. Of these, 14 were male and 34 were female, with ages ranging from 18 to 50 years. The selected participants were then thoroughly analyzed and randomly assigned to two groups. The control group (TDR) consisted of volunteers who danced in traditional dance studios after the pandemic, while the experimental group (SFM) consisted of volunteers who danced at home after the pandemic using digital devices such as the FITURE Magic Mirror Smart Fitness Mirror to track changes in mood and anxiety. The experimental group consisted of 24 participants (16 females and 8 males), and the control group consisted of 24 participants (18 females and 6 males). The mean age of the SFM participants was 25.83 ± 6.96 years, while the mean age of the TDR participants was 27.96 ± 7.30 years. Analysis of variance (ANOVA) revealed that there were no significant differences between the two groups of participants in terms of age, gender, job, type of dance, and skill level (*p* > 0.05).

**Figure 2:**
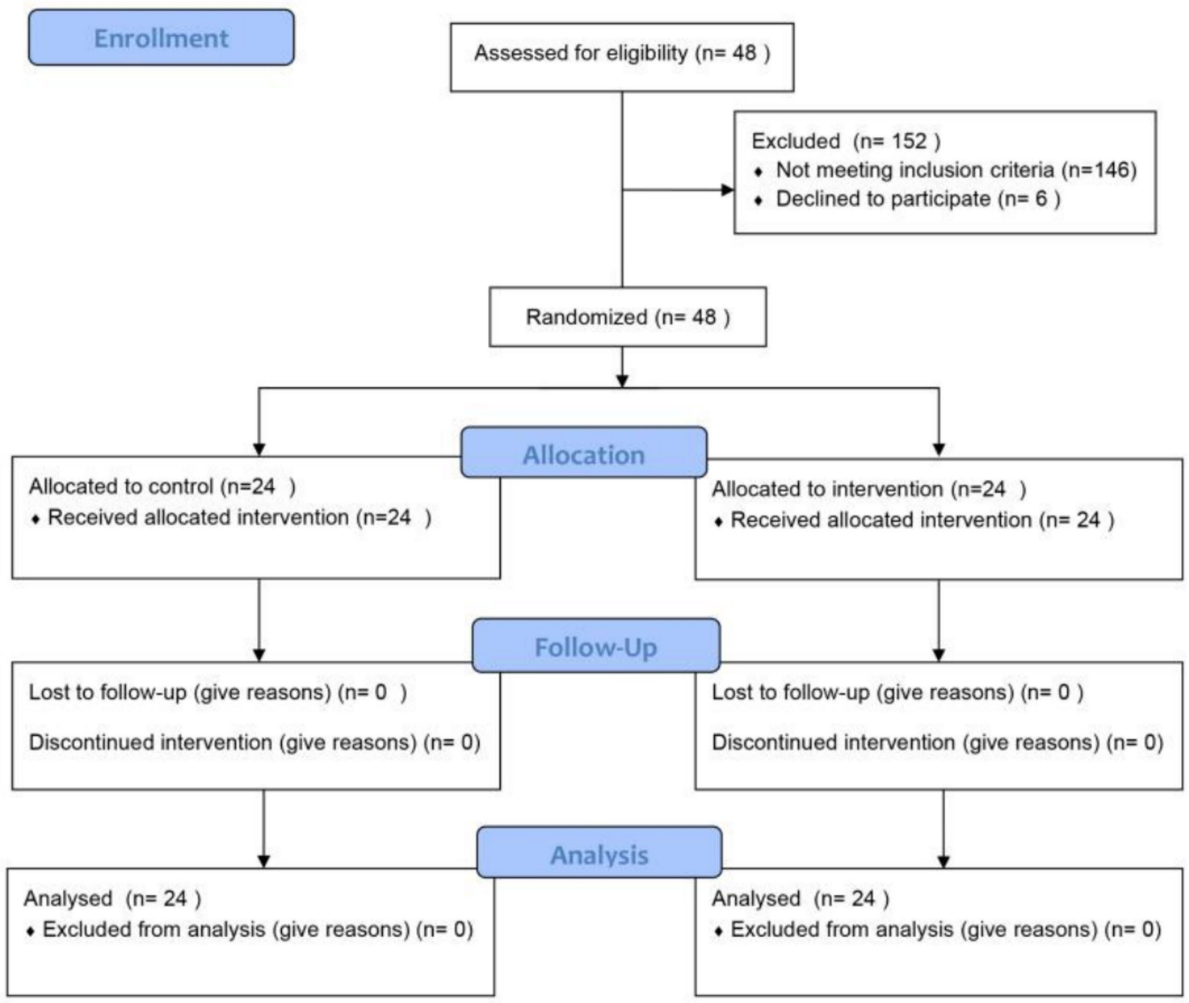
Consort flow diagram.

Inclusion criteria for participants were: self-assessment by participants via the Self-Rating Anxiety Scale (SAS) and clinical psychologist review of the scale results to screen a group of dancers with anxiety levels in the normal or mild range. Participants had not received psychoeducational or cognitive-behavioral training in the 3 months prior to the intervention. The CONSORT flow diagram of the study regarding the inclusion of the participants is shown in Figure 2.

After receiving the complete study instructions, all participants agreed to participate and signed a written informed consent form. The overall implementation framework included dance therapy architecture, experimental design, assessment, intervention design, and empirical testing. All participants volunteered for the experiment and reserved the right to withdraw at any time.

### 5.2 Measures

Using a randomized controlled trial (RCT) approach, this trial aims to assess whether the use of digital technology (e.g., smart fitness goggles) in dance therapy is effective in alleviating anxiety associated with pandemic isolation and compare it to traditional dance therapy.

#### 5.2.1 Intervention

This study required a baseline assessment of all participants prior to the experiment to understand their initial anxiety levels after pandemic isolation.

Both the TDR and SFM groups participated in a 3-month intervention. During this time, participants engaged in weekly self-directed dance training in a traditional dance studio or at home using smart fitness mirrors, depending on their group assignment. Participants in both groups were assessed at three time points: baseline (T0, before the start of the experiment), midterm (T1, 1 month post-intervention), and endpoint (T2, 3 months post-intervention). At each time point, participants’ mood changes were assessed using the Positive and Negative Affect Schedule (PANAS). There was no private communication between groups of participants.

#### 5.2.2 Mental health status

The Self-Assessment Scale for Anxiety (SAS) (Wang and Chi, 1984), which uses a 4-point scale to rate the frequency of symptoms as defined by the items, provides a comprehensive assessment of volunteers’ mental health and subjective feelings of anxiety.

The scale consists of 20 items, and its delineation values were drawn by distribution in a large sample of people, with a normal upper limit of 40 points for the total crude score of normal Chinese people. In this study, the severity delineation criteria were based on the current common hospital criteria (psychological testing software), while referring to the manual of commonly used psychological assessment scales (Dai, 2010) for delineation, with standardized scores of <50 points for no anxiety, ≥50 points for mild anxiety, ≥60 points for moderate anxiety, and ≥70 points for severe anxiety (Duan and Sheng, 2012).

This trial categorized participants into four groups based on the results of the pre-intervention questionnaire: Category A: normal range (total score less than 50), Category B: mild anxiety (total score between 50 and 60), Category C: moderate anxiety (total score between 60 and 70), and Category D: severe anxiety (total score greater than 70). A total of 48 volunteers in categories A and B were then randomly assigned in half by number to either an experimental group that used the smart fitness mirror (digital devices) at home (SFM Team) or a control group that was in a traditional dance room (TDR Team). The screening criteria for the volunteer samples in this experiment were: a total score <60, and volunteers who met the above criteria were selected as members of the experiment. The internal consistency of the overall scale was good with *α* = 0.821. The instrument proved to be a reliable and valid indicator of mental health in the general population (Dunstan and Scott, 2020; Liang et al., 2020).

#### 5.2.3 Measurement of positive and negative emotions

At each time point, participants’ mood changes were assessed using the Positive and Negative Affect Scale (PANAS). The scale consists of a positive affective subscale and a negative affective subscale, each containing five items, and is designed to measure psychological satisfaction in the general population. The scale is designed to be used as both a between-subjects and within-subjects measure of state or trait affect (Clark and Watson, 1994). Affective balance is calculated as a positive affect score minus a negative affect score, with an additional factor of 5, which ranges from 1 to 9. The use of assessment scales is a reasonable approach due to the wide variation in anxiety symptoms and the varying degrees of impact from the epidemic among dance enthusiasts in the group. The use of assessment scales is a reasonable approach due to the wide variation in anxiety symptoms and the varying degrees of impact from the epidemic among dance enthusiasts in the group (Humphries et al., 2023).

In order to control for additional variables, the experimental procedure ensured that the intervention was of the same duration for both groups, and was conducted twice a week for one and a half hours, with psychometric tests being administered before the experiment, after 1 month of the experiment, and after 3 months of the experiment, and that any additional interventions were scrupulously documented and excluded from the analyses. Also, experimenters were not allowed to perform additional traditional studio dance interventions. All questionnaire data were analyzed using a 2-group (traditional ballroom dance group, game group) × 3 times (pre-test, test, and post-test) repeated measures ANOVA. The internal consistency of the overall scale was good, *α* = 0.841.

#### 5.2.4 Satisfaction and propensity to use smart fitness mirrors

After the experiment, in order to better collect participants’ experiences and feedback, we also considered using a five-point Likert scale as well as a qualitative method: semi-structured interviews to complement the experiment.

The System Usability Scale questionnaire (SUS) assesses participants’ satisfaction and propensity to use smart fitness goggles and consists of six items. Measured on a 5-point Likert scale ranging from 1 = Strongly Disagree to 5 = Strongly Agree (Brooke, 1996), the questionnaire included questions such as: ‘This product relieves the anxiety of sociopaths and home-bound individuals like the participants’ and ‘I would be more likely to practice dance with this type of smart digital product than in a traditional dance studio/gym.’ The overall internal consistency of the scale was good, α = 0.806, and the instrument demonstrated validity and reliability in both dance and healthcare populations using digital technology devices (Mol et al., 2020; Martins et al., 2025).

### 5.3 Data collection

Participants in both the TDR and SFM groups were assessed using the Positive and Negative Affect Scale (PANAS) at baseline and at 1 and 3 months post-intervention (from July 2024 to October 2024) and were assessed using satisfaction and propensity questionnaires 1 month after the end of the intervention (November 2024), along with additional interviews. Participants were contacted prior to the assessment, given 1 week to complete the questionnaire, and reminded if they had not done so within the given period to ensure timely data collection.

### 5.4 Statistical analysis

We used methods such as density and quantile plots to assess the normal distribution of variables, supplemented by Shapiro–Wilk tests. Per-protocol analyses were performed (i.e., only participants who completed the entire study were included). Data were analyzed as the change from baseline (T0) to 1 month (T1) and 3 months (T2) for each group, determined by the mean difference and its 95% confidence interval (CI). For continuous variables, between-group differences at each time point were determined using independent-samples t-tests, while within-group differences over time were analyzed using paired t-tests. A two-way repeated-measures analysis of variance (ANOVA) was conducted to examine the interaction between group (SFM team vs. TDR team) and time (T0, T1, and T2) on PANAS scores for mood and anxiety levels. Psychological data was analyzed using SPSSAU(The SPSSAU project., 2024). The psychological data are presented as mean ±SD (standard deviation). Statistical significance was assumed for *p* < 0.05.

## 6 Research results

A total of *N* = 48 participants were included into analysis, samples from different groups did not show significant differences in gender, age, occupation, amateur/professional dancers. For more information, see Table 2.

**Table 2:** Baseline characteristics of all included participants.

Characteristics	Group (Mean ± SD)		t	p
	TDR (*n* = 24)	SFM (*n* = 24)		
Gender	1.25 ± 0.44	1.33 ± 0.48	−0.624	0.535
Age	1.92 ± 1.10	2.33 ± 1.20	−1.252	0.217
Occupation	3.08 ± 1.50	3.21 ± 1.89	−0.254	0.801
Amateur/Professional Dancer	1.46 ± 0.51	1.63 ± 0.49	−1.151	0.256
SAS Self-Assessment Scale Results (Present Mood State)	40.83 ± 9.33	41.33 ± 11.76	−0.163	0.871
Anxiety from new COVID-19 quarantine	2.00 ± 0.72	2.08 ± 1.14	−0.303	0.764

*p < 0.05 **p < 0.01.

The differences between the positive mood, negative mood and overall mood of the experimental and control groups were not significant between pre-experiment and post-experiment. After the intervention, the TDR group showed an increase in positive emotions and a decrease in negative emotions compared to the pre-intervention period, but none of the differences were significant (*p* > 0.05), as shown in columns TT0 & TT2 in Table 3. However, there was a significant improvement in the total score of the SFM group compared to the pre-experiment (*p* < 0.01), as shown in columns ST0 & ST2 in Table 3.

**Table 3:** Changes in positive mood, negative mood, and overall mood of experimental and control groups at each time point.

Group	Traditional dance room						Smart fitness mirror					
Time point	T0		T1		T2		T0		T1		T2	
Value	Means	SD	Means	SD	Means	SD	Means	SD	Means	SD	Means	SD
Positive	27.96	4.41	29.17	5.49	29.00	4.67	26.58	4.86	28.42	5.68	30.00	5.42
Negative	24.88	5.86	23.38	3.74	23.38	4.99	24.04	4.70	23.79	3.89	24.17	4.46
Result	1.46	0.83	1.17	0.38	1.25	0.61	1.79	0.98	1.42	0.83	1.21	0.59

T0, T1, and T2 represent the time after the quarantine of the pandemic, 1 month of treatment, and 3 months of treatment, respectively. Positive represents positive mood, negative represents negative mood, and result represents final overall mood.

### 6.1 Comparison of the differences between the experimental group and the control group before and after the trial

Table 3 shows the changes in the positive and negative mood results and factor scores of the test and control groups before and after the test, and it can be seen that the mean values of the positive mood increased in both groups, but the magnitude was larger in the SFM group; the mean values of the negative mood decreased in both groups; and in terms of the mean values of the results, both groups decreased, with the largest drop in the SFM group (the result of a high positive mood was recorded as 1, the result of an equal positive and negative mood was recorded as 2, for high negative mood is noted as 3). Comparison of the differences in pre and post changes showed (see Tables 4, 5 for details) that after 3 months of group dance healing, most of the anxiety in both groups improved, with the improvement in anxiety in the TDR group being more significant (*p* < 0.01) after 1 month as well as after 3 months of group dance healing.

**Table 4:** Comparison of the significant differences between different time points within the same group of the experiment.

Group	Traditional dance room						Smart fitness mirror					
Time point	TT0 & TT1		TT0 & TT2		TT1 & TT2		ST0 & ST1		ST0 & ST2		ST1 & ST2	
Value	t	*p*	t	p	t	p	t	p	t	p	t	p
Positive	−1.796	0.086	−1.401	0.174	0.228	0.822	−2.161	0.041*	−3.345	0.003**	−2.889	0.008**
Negative	1.889	0.072	1.429	0.166	0.000	1.000	0.343	0.734	−0.136	0.893	−0.857	0.400
Result	1.574	0.129	1.155	0.260	−0.811	0.426	3.245	0.004**	3.245	0.004**	0.000	1.000

*p < 0.05, **p < 0.01, ***p < 0.001; Replacing the Traditional Dance Room (TDR) group with T and S for the Smart Fitness Mirror group. For example, TT0 denotes the basal test phase of the TDR group. Table 5 follows the same structure.

**Table 5:** Comparison of significant differences at the same time within different groups of the experiment.

Time point	TT0 & ST0		TT1 & ST1		TT2 & ST2	
Value	t	*p*	t	*p*	t	*p*
Positive	1.199	0.243	0.581	0.567	−0.846	0.406
Negative	0.774	0.447	−0.399	0.694	−0.634	0.533
Result	−1.498	0.148	−0.44	0.664	0.225	0.824

The results of the experiment showed a significant improvement (*p* < 0.01) in the total score of SFM from pre-intervention (T0) (1.79 points ± 0.98 SD) to mid-intervention (T1) (1.42 points ± 0.83 SD) to post-intervention (T2) (1.21 points ± 0.59 SD). In contrast, the overall mood of TDR in the control group did not improve significantly (*p* > 0.05) from T0 (1.46 points ± 0.83 SD) to T1 (1.17 points ± 0.38 SD) to post-intervention (T2) (1.25 points ± 0.0.61 SD) (see Tables 2, 3).

The data in the T0, T1, and T2 columns in the traditional Dance Room group (hereafter referred to as the TDR group) in Table 3, as well as the TT0&TT1, TT0&TT2, and TT1&TT2 columns in Table 4, showed that after 3 months, although the control group’s positive mood increased and negative mood declined, the differences were not significant (*p* > 0.05).

### 6.2 Comparison of changes between the experimental group and the control group before and after the trial

**Figure 3:**
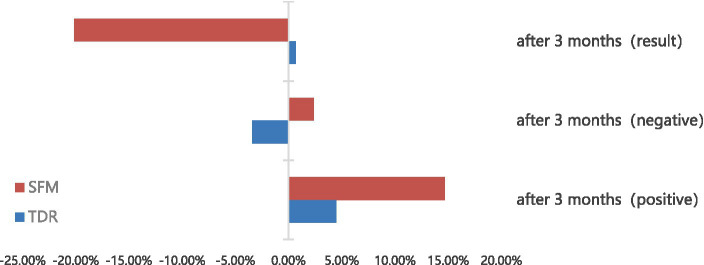
Comparison of the efficacy rate of each group after the trial.

As can be seen from Figure 3, the positive mood scores of both the experimental and control groups improved before and after the test, but the magnitude of the improvement varied, with the experimental group showing a particularly significant increase in positive mood (*p* < 0.001). In contrast to the control group, the negative scores of the experimental group increased, suggesting that the smart fitness mirrors did not give the volunteers more improvement in their negative emotions. In contrast, the outcome scores of the experimental group decreased after the trial, implying that the volunteers in the experimental group had improved their anxiety (the outcome was recorded as 1 for high positive mood, 2 for equal positive and negative moods, and 3 for high negative moods), and the improvement was also highly significant (*p* < 0.001). In contrast, outcome scores in the control group increased after the trial, and improvement was not significantly different (*p* > 0.05).

Remarks: Efficacy rate = (post-treatment score - pre-treatment score)/pre-treatment score. Generally speaking, the absolute value of the efficacy rate ≧50% is considered effective, and ≧25% is considered effective (the positive and negative values of the efficacy rate are analyzed on a case-by-case basis).

From the data, the absolute value of the efficacy rate of the two groups did not exceed 25%, both in terms of the outcome and the factor scores. The positive efficacy rate for the experimental group and the negative efficacy rate for the outcome indicate that the anxiety of the experimental group’s personnel was reduced after the intervention. However, the negative efficacy rate was positive, indicating that some anxiety was not alleviated. In contrast, the TDR group showed a positive resultant efficacy rate, although the positive mood was positive and the negative mood was negative. This suggests that in the control group, some members of the group received increased anxiety symptoms from the pandemic isolation than in the absence of the intervention treatment, anxiety was not better alleviated, and interpersonal relationships became more sensitive.

### 6.3 Intervention effect of the experimental group

Of the members who participated in the art healing trial using the Digital Intelligent Dance device, all volunteers’ anxiety improved and no one became worse (see Table 6 for details). From the results, 26% of the volunteers had an extremely significant intervention (*p* < 0.001), changing from negative to positive, and 1 volunteer had a significant change from high negative mood to equal positive and negative mood, while 70% of the volunteers did not have a significant change, but all of them tested high in positive mood. In terms of the numerical change in positive mood, more than half of the volunteers showed an increase in positive mood, with 12% of the volunteers showing an extremely high increase in positive mood (values >10) and 17% of the volunteers showing a significant increase in positive mood, however, 25% of the volunteers showed a decrease in positive mood instead. In terms of the change in the value of negative emotion, 17% of the volunteers had no change in the before and after values, 37% of the volunteers had a decrease in negative emotion, of which 12% of the volunteers had a significant effect on the decrease in negative emotion. And nearly half of the volunteers’ negative emotions increased instead, with no significant trend.

**Table 6:** Intervention effects in the experimental group.

ID	Values of positive mood change	Values of negative mood change	Changes in overall mood
1	−2	4	No change
2	10	−6	Negative to positive
3	15	9	No change
4	3	0	No change
5	0	0	No change
6	−1	−1	No change
7	3	0	Negative to positive
8	3	0	No change
9	2	3	No change
10	1	4	No change
11	6	−4	Negative to positive
12	1	−1	No change
13	3	−2	Negative to positive
14	13	−9	Negative to positive
15	−1	1	No change
16	9	−9	Negative to positive
17	1	1	No change
18	0	3	No Change
19	−1	−3	Negative to equal
20	5	2	No change
21	6	5	No change
22	−2	−3	No change
23	−3	1	No change
24	11	8	Equal to positive

The numbers are all for the values of mood after 3 months of treatment minus the values of mood after quarantine of the epidemic.

As shown in Table 7, in the experimental group, the mean value of positive mood before the intervention was 26.58, and the mean value of overall mood was 62.68. After participating in the three-month art healing program, the mean value of positive mood increased to 28.42 and then finally to 30.00, while the mean value of overall mood decreased to 1.21, a reduction of 32.40%. These results indicate that the anxiety of the volunteers in the experimental group was significantly reduced through art healing. Additionally, the smart fitness mirror had a notable therapeutic effect on the anxiety caused by the pandemic quarantine and was more effective than dancing in a traditional dance studio.

**Table 7:** Changes in positive symptoms.

Group	Pre-test means	Under-test means	Post-test means	Rate of increase
Positive	26.58	28.42	30.00	12.87%
Result	1.79	1.42	1.21	−32.40%

*p < 0.05, **p < 0.01, ***p < 0.001.

### 6.4 Results of the interviews

After the completion of the trial, each volunteer was interviewed, and some of the interviews, as well as the results, are shown in Table 8.

**Table 8:** Likert scale ANOVA results.

ANOVA results				
Questions	Group (Mean ± standard deviation)		*F*	*p*
	TDR (*n* = 24)	SFM (*n* = 24)		
Q87_1.I am willing to use smart dance devices when I feel emotionally unsettled.	3.63 ± 1.35	2.00 ± 0.98	22.910	0.000**
Q88_2.The smart AI dance coach helps me get into a learning state better than a traditional dance teacher.	4.21 ± 1.02	2.13 ± 1.23	40.896	0.000**
Q89_3.Smart dance devices help alleviate the anxiety of people like me who have social anxiety or prefer staying at home.	3.54 ± 1.41	1.96 ± 0.75	23.488	0.000**
Q91_5.I prefer using smart digital products to practice dance compared to traditional dance rooms/gyms.	4.04 ± 1.08	1.88 ± 0.80	62.317	0.000**
Q92_6.I feel anxious about others noticing my body in traditional dance rooms, but using the smart fitness mirror at home alleviates this anxiety.	3.83 ± 1.40	1.92 ± 0.83	33.153	0.000**

*p < 0.05 **p < 0.01.

We used one-way ANOVA to study the two groups’ perceptions of digital smart products such as the smart fitness mirror. From the results, both groups of volunteers showed significance (*p* < 0.05) for the following questions, implying that the different groups of samples are different for all.

Both groups of volunteers were willing to use smart dance devices when they were upset, and those who had used smart fitness mirrors (mean 2.00) were more willing to use smart dance devices to ease their uneasy moods compared to those who only practiced in traditional dance studios (mean 3.62). In the choice of AI dance instructors as well as teachers in traditional dance studios, volunteers in the SFM group (mean 2.12) felt that the AI dance instructors could help them get into a better dancing state, whereas volunteers in the TDR group (mean 4.21) still felt that the real-life teachers were more approachable and able to solve problems at any time. Whereas both groups of volunteers felt that smart dance devices like smart fitness mirrors could somewhat alleviate the social anxiety left over from the pandemic, both also felt that home smart fitness mirrors could avoid the problem of body image anxiety being exposed to the general public. As a result, both groups of volunteers preferred to continue their dance training in their original respective ways. Dancers in the SFM group believed that smart devices such as smart fitness mirrors were easy to use, flexible in terms of time, and alleviated the cost of traveling and class enrollment, which could alleviate the uneasy emotions of body anxiety and social anxiety. Dancers in the TDR group believed that traditional studio teachers can better instruct dancers face-to-face on standardized movements, are more interactive, have a better sense of dance atmosphere, and are more able to keep dancers focused.

## 7 Discussion

From the results of this study, it is clear that the quarantine of the pandemic did leave people with anxiety, and the body image anxiety, as well as the social fear anxiety mentioned in this paper, are just some of the high prevalence types of anxiety among many others. Anxiety is currently a high-prevalence psychological disorder in our population, with prevalence rates of about 20–30% and 15–25%, respectively (Shi et al., 2020; Chen et al., 2023; Bin et al., 2024). The SAS self-assessment scale as well as the PANAS Positive and Negative Scale can directly assess people’s anxiety, but the symptoms of anxiety are complex and diverse, and some of the symptoms of other factors may be related to it, such as obsessive-compulsive disorder, hostility, phobias, sensitivity to relationships, and somatization, etc., Anxiety often triggers interpersonal sensitivity, and this psychological stress in turn manifests itself through somatic symptoms (Tellawi et al., 2016).

The trial found that the findings aligned with those of Moratelli et al. (2023). Age, occupation, and gender factors had no significant effect on improving anxiety remaining after pandemic isolation. Being a professional dancer or not did not make the effect of anxiety improvement more significant (*p* > 0.05). The status of filling out the positive and negative mood scale shows that all volunteers improved their mental health after participating in the 3-month dance healing process, but there were differences between different groups, with the Smart Fit Mirror group having a more significant effect (*p* < 0.001). However, close to half of the volunteers in the SFM group’s negative emotions increased instead after the trial, which was not a significant trend but indicated that the effect of the Smart Fitness Mirror on the alleviation of negative emotions still needs to be improved, and was not as effective as that of positive emotion promotion. This finding resonates with the discussion on integrating technology into mental health treatment, emphasizing the potential of digital tools and the need for careful implementation to avoid negative effects (Bond et al., 2023; Olawade et al., 2024; Coelho et al., 2025).

Post-trial interviews revealed a generally positive attitude toward digital dance products. Participants acknowledged that these devices could alleviate social anxiety stemming from the pandemic and address body image anxiety when exposed to the public. However, individuals unfamiliar with such products expressed a preference for traditional dance studio environments, citing the value of face-to-face interactions and skepticism toward AI dance instructors.

Although this study found that dance healing is effective in relieving anxiety, more than half of the volunteers failed to achieve significant therapeutic effects within a short period due to the limited duration of the intervention (3 months). This result aligns with existing literature emphasizing the importance of intervention length (Salihu et al., 2021). Therefore, further relevant studies need to extend the intervention time, increase the sample size, and explore the long-term efficacy of smart fitness mirrors in individuals with different anxiety levels. In addition, AI dance coaches currently still suffer from insufficient human interaction in their interactions with users, and future research should aim to improve technological interactions in order to increase users’ trust and dependence on digital devices. Additionally, the lack of human interaction with AI dance coaches may limit user trust and engagement, pointing to the need for enhancing technological interactions to foster better human-computer synergy. Future AI dance instructors will likely have enhanced language comprehension, refined emotional perception, and more flexible physical coordination. At that time, AI coaches can interact with students in a deeper and more personalized way, providing more targeted guidance and companionship. This study not only provides new ideas for the field of dance healing, but also provides valuable references for the direction of human-computer interaction and emotional computing in the field of digital intelligence.

### 7.1 Practical significance

Our study focuses on analyzing whether the integration of digital technology into its method is more effective in treatment outcomes in the context of the prevalence of dance therapy. The experimental results verified the unique positive significance of dance therapy incorporating digital somatic technology in relieving anxiety, i.e., this therapy has stronger intervention advantages in relieving body image anxiety and alleviating social fears compared to dance therapy in traditional dance studios. More importantly, AI coaches are unable to provide the emotional support, encouragement and psychological guidance that real coaches can provide, which will greatly affect learners’ motivation and sense of achievement. Therefore, the incorporation of digital technology can be an important complementary tool to traditional dance therapy.

In the context of digital technology, AI dance coaches provide users with personalized and efficient dance teaching through motion capture, data analysis and other technologies, filling geographical and economic gaps. However, AI dance coaches currently lack the ability to provide the emotional support, encouragement, and psychological guidance offered by human coaches, and tend to generate similar styles of dance movements, leading to homogenization of dance therapy styles and weakening the diversity of dances, which can greatly affect the motivation and sense of achievement of those being treated. All of these issues require us to think deeply and develop appropriate measures while enjoying the convenience of AI (Liu, 2024).

This paper will provide a literature base for future research on digital technologies, especially AI dance coaches, and in particular, the integration of somatically interactive digital technologies into dance healing to reach the goal of online dance therapy with no consideration of the user’s dance expertise, thus alleviating the symptoms of body image anxiety and social anxiety that are left over from the post-pandemic era.

### 7.2 Foresight

In summary, Digital Dance Healing should consider human-machine synergy even more in the future, combining AI coaches with real coaches to take advantage of each other’s strengths. AI coaches are responsible for providing real-time, efficient and personalized basic teaching and feedback, while real coaches are responsible for giving emotional value, psychological guidance, and imparting an understanding of dance culture. Expanding the diversity of training data for AI coaches is also a priority, including different styles and cultures of dance to avoid homogenization. Technicians should also think about how to incorporate cultural elements into AI algorithms so that the AI can convey the cultural connotations behind the dance. We have also summarized a new approach to dance healing based on digital technology based on the research done, as shown in Table 9.

**Table 9:** A new approach to dance healing based on digital technology.

Emotional condition	Recommended approach	Details
High Positive PANAS (Positive Emotion)	Traditional Studio Classes	Enroll in a dance studio for in-person classes, assuming the same dance fundamentals.
High Negative PANAS (Negative Emotion)	Digital Smart Dance Equipment (Home)	Use smart fitness mirrors or similar digital dance equipment at home.
Large Difference Between Negative and Positive (≥ 5 points)	Combination of Digital Equipment & Traditional Classes (Early Stage)	Use digital equipment at home along with traditional studio classes at the start.
Equal Positive and Negative Emotions	Dancer’s Preference	The choice can be made based on the dancer’s personal preference.

## 8 Conclusion

Overall, the dance art therapy was significantly effective in alleviating the dancers’ anxiety left over from the quarantine of the pandemic, especially body image anxiety as well as social anxiety, and the dancers’ positive emotions were enhanced. Through randomized scientific control, the smart fitness mirror group was found to be more effective than the traditional studio group in relieving body image anxiety as well as social anxiety. The study also found that by using digital devices such as smart fitness mirrors, dancers were able to better express and understand their emotions, enhancing self-awareness and emotion regulation.

However, the current study still has some limitations, such as a relatively small sample size due to the geographical distribution of subjects. In future studies, we will further expand the sample size and track it over time. In particular, we will overcome geographical limitations and develop new measurement approaches from various perspectives to refine the findings and provide a more comprehensive and in-depth mental health assessment for dancers. This will help obtain stronger evidence supporting the integration of digital technology into dance art therapy.

## Data Availability

The original contributions presented in the study are included in the article/supplementary material, further inquiries can be directed to the corresponding author.
